# Prism Design for Spectral Flow Cytometry

**DOI:** 10.3390/mi14020315

**Published:** 2023-01-26

**Authors:** Zixi Chao, Yong Han, Zeheng Jiao, Zheng You, Jingjing Zhao

**Affiliations:** 1 Department of Precision Instruments, Tsinghua University, Beijing 100084, China; 2 Department of Structural Biology, School of Medicine, Stanford University, Stanford, CA 94305, USA

**Keywords:** flow cytometer, micro-flow cytometer, spectrometry, prism, dispersion

## Abstract

Flow cytometers are instruments used for the rapid quantitative analysis of cell suspension. Traditional flow cytometry uses multi-channel filters to detect fluorescence, whereas full-spectrum fluorescence based on dispersion detection is a more effective and accurate method. The application of various dispersion schemes in flow cytometry spectroscopy has been studied. From the perspective of modern detectors and demand for the miniaturization of flow cytometry, prism dispersion exhibits higher and more uniform light energy utilization, meaning that it is a more suitable dispersion method for small flow cytometers, such as microfluidic flow cytometers. Prism dispersion designs include the size, number, and placement of prisms. By deducing the formula of the final position of light passing through the prism and combining it with the formula of transmittance, the design criteria of the top angle and the incident angle of the prism in pursuit of the optimum transmittance and dispersion index can be obtained. Considering the case of multiple prisms, under the premise of pursuing a smaller size, the optimal design criteria for dispersion system composed of multiple prisms can be obtained. The design of prism dispersion fluorescence detection was demonstrated with a microfluidic flow cytometer, and the effectiveness of the design results was verified by microsphere experiments and practical biological experiments. This design criterion developed in this study is generally applicable to spectral flow cytometers.

## 1. Introduction

A flow cytometer is a life science instrument that can rapidly and quantitatively analyze the physical and chemical information of multiple parameters of each individual cell in a cell population and distinguish or select target cells according to the characteristic information. The research object of flow cytometry is not only limited to cells but also contains various kinds of biological particles, such as bacteria, parasites, viruses, microorganisms, parasites, proteins, or other molecules [1]. Flow cytometers have the advantages of high throughput and high accuracy and are widely used in medical, biological, and other fields.

At present, fluorescence detection is the main method of cell analysis via flow cytometry. Traditionally, multi-channel filter detection has been the main way to detect fluorescence at different wavelengths [2,3,4,5]. However, the fluorescence spectra of various dyes have overlapping problems. When the overlap ratio is too high, the results based on the compensation of several channels are not accurate or reliable. Comprehensive solutions based on full-spectrum data have more information (analysis basis) and higher accuracy and reliability [6,7]. For an experiment involving a variety of different fluorescence dyes, the workload of experiments that require mutual compensation of fluorescein will increase by the order of square, and the consumption of experimental samples will be huge, making it difficult to carry out experiments involving super multiple types of fluorescence. Spectral measurements can be combined with integrated and comprehensive spectral algorithms; thus, these experiments can be realized consuming low amounts of samples and a limited experimental workload. Therefore, spectral flow cytometry is a more advanced and innovative scheme than traditional flow cytometry.

As early as 1979, C.G. Wade from the University of Texas tried to use spectroscopic instruments to measure the spectrum of fluorescence emitted by cells [7]. However, due to the performance of vidicon technology, it was difficult to achieve high signal-to-noise ratio fluorescence spectrum detection for a single cell. In 1986, Harald B. Steen from Norsk Hydro’s Institute for Cancer Research introduced a grating dispersion system and photomultiplier tube (PMT) for fluorescence spectrum measurements in flow cytometry [8,9]. This study measured the average spectrum of cells, not the spectrum of individual cells. This is not the function of spectral flow cytometers in the present sense. In 1990, Tudor N. Buican from the Los Alamos National Laboratory used a Fourier spectrometer to address the difficulties that array detectors exhibited in achieving high-speed signal acquisition at that time [10]. However, compared with the fluorescence time in conventional flow cytometers, this principle of spectral measurement still requires a fluorescence signal that lasts for a long time, which greatly limits the detection flux. In 1996, Charles L. Asbury from Norsk Hydro’s Institute for Cancer Research used a monochromator and a single-tube PMT to measure different single spectral bands [11]. It failed to measure the spectral information of each band of each cell at the same time. In the same year, M.R. Gauci from Macquarie University tried to measure spectra based on prism dispersion, but the detector used was an array photodiode, and the detection ability was poor for weak fluorescence [12]. The performance limit of the electronic system reading data in that year also affected the detection flux. In addition, the specific design of the prism was simply using a single prism, without in-depth research. In 2006, Gregory Goddard from Los Alamos National Laboratory finally realized the spectral flow cytometer scheme that can be used to detect the flux of conventional flow cytometers under the grating dispersion scheme by using CCD array as the detector [13]. However, it still has the problem of poor weak light detection ability. In 2012, Gérald Grégori from Purdue University used 32-array PMTs and realized a large instrument for the spectral measurement of flow cytometer fluorescence signals based on grating dispersion and compared its effect with that of traditional flow cytometers [14]. In 2015, Sony and Kyoto University cooperated in a study, which still used 32-array PMT, further developing the single prism dispersion scheme into prism groups and forming a commercial large-scale spectral flow cytometer [15]. In 2017, Cytek Biosciences proposed a design for compact reciprocating reflection filter spectrum detection. This design could also be used to realize spectral flow cytometers [16].

Research into spectral instruments and spectral flow cytometry is mature and has been commercialized. However, general spectral instruments have low light energy utilization rate and low linear dispersion rates; thus, it is difficult to adapt array PMTs or other photoelectric detection devices to smaller sizes, so they are not suitable for spectral flow cytometers. In the field of spectral flow cytometry, the miniaturization problem is not generally considered at present. Existing spectral flow cytometer products are large-scale instruments, and they are generally combined with complex optical systems, which are very difficult to assemble. This affects the portability of the instrument and its applicability on certain occasions. For existing spectral flow cytometry designs, there is also a lack of a universal model, which is not conducive to subsequent research.

In existing spectral flow cytometry research, the detector and electronic system were the core technical bottlenecks in the early years; therefore, the dispersion design mainly aimed to meet the requirements of these parts. At present, array PMT devices with higher signal-to-noise ratio and stronger weak light detection ability have emerged. These technologies have exhibited great progress, so the dispersion design scheme can also have greater freedom of choice. The dispersion schemes involved in previous studies and instrument products include grating dispersion, prism dispersion, Fourier spectrometry, and basic filtering schemes. The Fourier spectrometer scheme appeased fluorescence detection systems in the early years, and it is difficult to achieve the same detection flux as the general flow cytometer. The light filtering scheme has the problem of low light energy utilization. Grating dispersion has been studied extensively, but from the physical properties of grating, it can only design a suitable blaze angle for a specific wavelength. The light energy utilization ratio of the wave band outside the flare angle will decline significantly. However, in flow cytometers, especially for the commonly used 488 nm laser, the corresponding fluorescence has a wide wavelength range; thus, the effect is poor when realizing demand for the simultaneous detection of wide band spectra of flow cytometers. As for the existing prism dispersion scheme, in addition to the problem of light energy utilization, its design does not consider the overall size, dispersion rate, and other indicators required by the miniaturization of the instrument and has not been optimized in this regard.

In view of the above situation, this study analyzed the specific application of prism dispersion in flow cytometry fluorescence detection, details the design criteria through analysis, and provides examples of a design scheme.

## 2. Modeling

As analyzed above, this study aimed to derive the design theoretical model of a prism dispersion system in a flow cytometer. The first step was to analyze the single prism model. Then, the optimal multi-prism dispersion system was optimized based on the light energy utilization, overall size, and dispersion rate.

**Figure 1:**
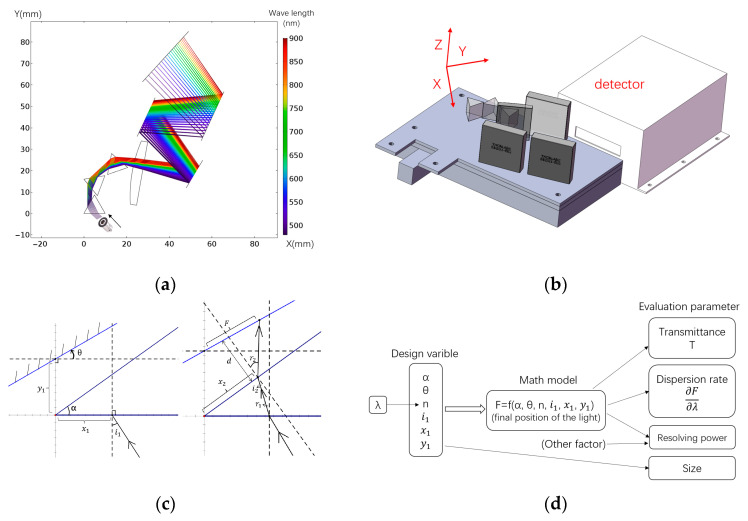
Prism dispersion model: (**a**) schematic diagram of optical path structure; (**b**) optical path structure layout; (**c**) geometric model and variable name of single prism; (**d**) logic diagram of the mathematical model.

The model prism dispersion system is shown in Figure 1a,b. A parallel beam passes through a few prisms for dispersion, then the dispersed lights pass through a convex lens and are reflected by several mirrors to minimize the system volume; eventually, the lights are focused onto the PMT array. The main targets for optimization of the dispersion system are to maximize the dispersion rate or spectrum resolution, to maximize the transmittance for a high light efficiency, and to minimize the system volume. Specific requirements were tentatively set as a linear dispersion rate of 0.2 mm/nm, resolution of 10 nm, and transmission characteristics of 30% (insertion loss −5 dB), and the longest side of the system is 10 cm.

The geometric model of a single prism and the related variables are shown in Figure 1c. The model can be unified as *F* = *f*(α, *θ*, *n*(*λ*), $i_{1}$, $x_{1}$, $y_{1}$), where *f* is the relation function calculated by geometrical optics, *λ* is the wavelength of the beam, and *n* is the refractive index and affected by the wavelength [17,18,19]. For the beam with a certain width and containing different wavelengths, the result of *F* will be multiple continuously distributed optical bands. Each pair of ($x_{1}$, *λ*) ordered numbers corresponds to an *F*. The linear dispersion rate of the system is ∂F/∂λ=(∂F/∂n)(dn/dλ). As for the resolving power, resolution is affected and limited by many factors. If the beam is assumed to have a certain width, xΔ, then to separate the two wavelengths, λΔ, the following two conditions need to be satisfied: first, the distance between the two wavelengths eventually separated is not less than the smallest scale of the detector pixel; second, the two wavelengths separated do not overlap in space (crosstalk). For the former condition, if the minimum size of the detector pixel is *D*, then D≤λΔ(∂F/∂λ). For the latter condition, *f*(*α*, *θ*, *n*(*λ*), $i_{1}$, x1+xΔ, $y_{1}$) ≤ *f*(*α*, *θ*, *n*(*λ* + λΔ), $i_{1}$, $x_{1}$, $y_{1}$). In the case of small variation, it can be reduced to xΔ(∂F/∂x1)≤λΔ(∂F/∂λ). Thus, the minimum wavelength resolution λΔ=max(D,xΔ(∂F/∂x1))/(∂F/∂λ), where the max function represents the maximum value between the two. According to the definition of resolution, R=λ/λΔ; thus, R=λ(∂F/∂λ)/max(D,xΔ(∂F/∂x1)). As for light transmission characteristics, the light loss, *T*, caused by reflection, assuming natural light, then the transmittance which only considers the reflection loss is the product of the transmittance of two optical surfaces, and then:

(1) $$\begin{array}{l} T=\left(1-\left({\left(\sin(i_{1}-r_{1})/\sin(i_{1}+r_{1})\right)}^{2}+{\left(\tan(i_{1}-r_{1})/\tan(i_{1}+r_{1})\right)}^{2}\right)/2\right)\\ {}*\left(1-\left({\left(\sin(i_{2}-r_{2})/\sin(i_{2}+r_{2})\right)}^{2}+{\left(\tan(i_{2}-r_{2})/\tan(i_{2}+r_{2})\right)}^{2}\right)/2\right) \end{array}$$

The other factors affecting the transmittance include the scattering of the interface and the scattering and absorption of the material inside the prism. For ordinary optical materials, the scattering and absorption of materials inside the prism are very weak and can be neglected. The scattering of the interface may be too large to be ignored, but considering that the scattering of the interface is diffuse reflection, it is random, i.e., isotropic. Therefore, there is no relationship between the degree of impact and the parameters which need to be designed. For the design of a single prism, it is not necessary to consider this factor. In conclusion, the complete math model is shown in Figure 1d.

### 2.1. Single Prism

For a single prism, the result is ($x_{2}$, $r_{2}$) in Figure 1c. We can derive $x_{2}=x_{1}\left(\cos\left(\left(\sin i_{1}\right)/n\right)\right)/\cos(\alpha -\arcsin\left(\left(\sin i_{1}\right)/n\right))$ (regard as $x_{2}=kx_{1}$, where $k=\left(\cos\left(\left(\sin i_{1}\right)/n\right)\right)/\cos(\alpha -\arcsin\left(\left(\sin i_{1}\right)/n\right))$), and $r_{2}=\arcsin(\mathrm{nsin}(\alpha -\arcsin\left(\left(\sin i_{1}\right)/n\right)))$. Considering the general situation, the receiving screen of the light is basically perpendicular to the light emitted. Thus, the position of the light on the receiving screen is considered as $F=x_{2}+dr_{2}$, where *d* is the distance from the position of the light-emitting prism to the receiving screen.

The linear dispersion of the system should be ∂F/∂λ. Sum *D* is a fixed quantity; thus, $\partial F/\partial \lambda =\left(\partial k/\partial n\right)\left(dn/d\lambda \right)x_{1}+\left(\partial r_{2}/\partial n\right)\left(dn/d\lambda \right)d=\left(\left(\partial k/\partial n\right)x_{1}+\left(\partial r_{2}/\partial n\right)d\right)\left(dn/d\lambda \right)$. Then:

(2) $$\begin{array}{l} \partial k/\partial n=\left(\sin i_{1}\sin\left(\left(\sin i_{1}\right)/n\right)\right)/\left(n^{2}\cos(\alpha -\arcsin\left(\left(\sin i_{1}\right)/n\right))\right)\\ +\left(\sin i_{1}\sin(\alpha -\arcsin\left(\left(\sin i_{1}\right)/n\right))\cos\left(\left(\sin i_{1}\right)/n\right)\right)/\left(n^{2}\cos^{2}(\alpha -\arcsin\left(\left(\sin i_{1}\right)/n\right))\sqrt{1-{\left(\left(\sin i_{1}\right)/n\right)}^{2}}\right) \end{array}$$

(3) $$\begin{array}{l} \partial r_{2}/\partial n=\left(\sin(\alpha -\arcsin\left(\left(\sin i_{1}\right)/n\right))+\left(\sin i_{1}\cos(\alpha -\arcsin\left(\left(\sin i_{1}\right)/n\right))\right)/\left(n\sqrt{1-{\left(\left(\sin i_{1}\right)/n\right)}^{2}}\right)\right)\\ /\sqrt{1-n^{2}\sin^{2}(\alpha -\arcsin\left(\left(\sin i_{1}\right)/n\right))} \end{array}$$

Therefore, ∂F/∂λ is a linear combination of ∂k/∂n and $\partial r_{2}/\partial n$ with the limitations of: $\alpha >\arcsin\left(\left(\sin i_{1}\right)/n\right)$ and $\mathrm{nsin}(\alpha -\arcsin\left(\left(\sin i_{1}\right)/n\right))<1$. Then, we try to find the best design of *α* and $i_{1}$.

**Figure 2:**
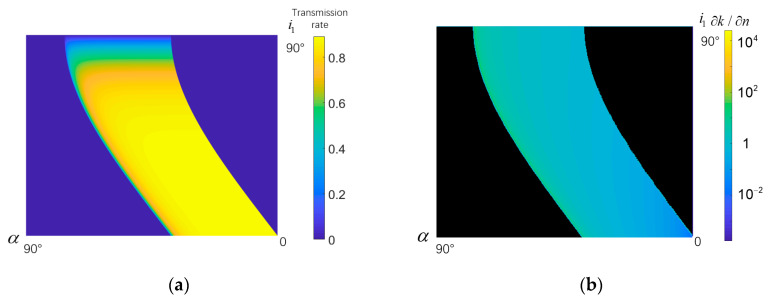

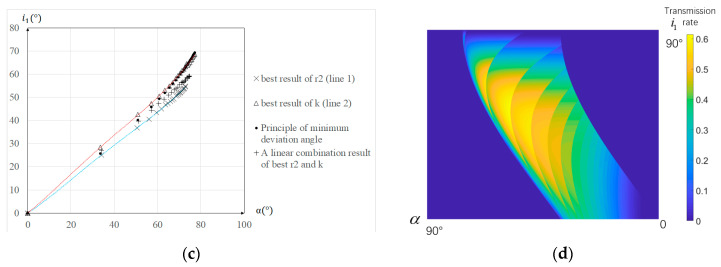
Prism design: (**a**) single prism transmission rate; (**b**) key coefficient of dispersion rate of single prism; (**c**) single prism parameter design scope (see above for specific explanation); (**d**) multiple prisms transmission rate.

In the available range (T>0) of Figure 2a, we look for “the highest value of dispersion under a certain lower limit of transmittance”, and “which *α* and $i_{1}$ can reach that best value (highest dispersion)”. Figure 2b shows ∂k/∂n, which is one of the factors that inflects dispersion. Figure 2c shows which point should be chosen according to the transmittance required by the design. In the figure, the “best result of $r_{2}$” means that the values of *α* and $i_{1}$ can reach the highest $\partial r_{2}/\partial n$ under a certain lower limit of transmittance, while the “best result of *k*” means that the values of *α* and $i_{1}$ can reach the highest ∂k/∂n under a certain lower limit of transmittance. If we want a higher transmittance, we can choose the point on the lower left side of the line (and the dispersion rate will be lower); if the requirement for transmittance is normal, then the point on the upper right side of the line should be chosen, so that the dispersion rate will be higher. In fact, the dispersion rate is determined by the results of these two linear combinations. The coefficients of linear combination are $x_{1}$ and *d*. Therefore, the actual optimal location is located in the narrow angular region surrounded by the two lines (the blue line and the red line in Figure 2c). Which point to choose depends on the requirement of transmittance and the relative relationship between $x_{1}$ and *d*. For example, the ‘+’ scatter points in the figure represent the different values of *α* and $i_{1}$ determined by different lower transmittance limits under a linear combination of $x_{1}$ and *d*.

In prism designs, the principle of the minimum deviation angle refers to the requirements in prism design where the light of the central wavelength inside the prism and the two sides of the prism form an isosceles triangle to make the whole optical path symmetrical. This method has the advantages of reducing aberration, having no additional transverse magnification, a small prism size, and being easy to design and adjust. Here, we also draw the values of *α* and $i_{1}$, which conform to the principle of minimum deviation angle in Figure 2c. It can be seen that under the higher transmittance requirements, *α* and $i_{1}$, which conform to the principle of minimum deviation angle, are located in the optimal value region. If the transmittance requirement is low and the apex angle is large, the *α* and $i_{1}$ which conform to this principle will exceed this region. If *α* and $i_{1}$ conform to this principle are in this region, we can select the specific proportion of $x_{1}$ and *d* to not only ensure that the design results have the best dispersion rate, but also conform to the minimum deviation angle principle in the next design. Specific follow-up design steps are concluded as follows:

1. Identify at least what transmittance is required;
2. For a definite lower limit of transmittance, draw a short line across the corner area and connecting the two sides of the angle in the corner area between line 1 and line 2 in Figure 2c (by changing the ratio of $x_{1}$ and *d*). There is a point of intersection between this line and the line which conforms to the principle of minimum deviation angle;
3. Design a ratio of $x_{1}$ and *d* (i.e., the ratio of $x_{1}$ and *d* at the intersection point in the second step), so that the value line of *α* and $i_{1}$ in this ratio passes through the intersection point above.

### 2.2. Multiple Prisms

For multiple prisms, the arrangement of relative positions between prisms should also be considered. We can use the above model, which is directly received by the light screen after passing through the prism, to nest the results.

In the following derivation, $i_{1}^{\left(j\right)}$ represents the incident angle of the entry prism in the *j*th prism model. The representation of other variables is similar. $F_{0}^{\left(j\right)}$ represents the position of the top angle of the prism corresponding to *F* in the *j*th prism model relative to the previous prism model. For example, for the second prism, the prism transfer equations $i_{1}^{\left(2\right)}=\alpha^{\left(1\right)}-\theta^{\left(1\right)}-r_{2}^{\left(1\right)}$ and $x_{1}^{\left(2\right)}=F(\alpha^{\left(1\right)},\theta^{\left(1\right)},n^{\left(1\right)},i_{1}^{\left(1\right)},x_{1}^{\left(1\right)},y_{1}^{\left(1\right)})-F_{0}{}^{\left(2\right)}$ can be obtained. Generally, $i_{1}^{\left(j\right)}=\alpha^{\left(j-1\right)}-\theta^{\left(j-1\right)}-r_{2}^{\left(j-1\right)}$ and $x_{1}^{\left(j\right)}=F(\alpha^{\left(j-1\right)},\theta^{\left(j-1\right)},n^{\left(j-1\right)},i_{1}^{\left(j-1\right)},x_{1}^{\left(j-1\right)},y_{1}^{\left(j-1\right)})-F_{0}{}^{\left(j\right)}$. Finally, assuming that there are *P* prisms in total, on the final receiving plane, the result (spot position) is as follows: $F^{\left(p\right)}=F_{p}(i_{1}^{\left(1\right)},x_{1}^{\left(1\right)},\alpha^{\left(1\right)},\theta^{\left(1\right)},n^{\left(1\right)},y_{1}^{\left(1\right)},F_{0}{}^{\left(2\right)},\alpha^{\left(2\right)},\theta^{\left(2\right)},n^{\left(2\right)},y_{1}^{\left(2\right)}\ldots F_{0}{}^{\left(p\right)},\alpha^{\left(p\right)},\theta^{\left(p\right)},n^{\left(p\right)},y_{1}^{\left(p\right)})$. In general, in the optical path arrangement, the total deflection angle should not exceed 180 degrees. Otherwise, the incident light and the outgoing light will intersect. It is easy to determine the deflection angle of a single prism: $\delta =i_{1}+r_{2}-r_{1}-i_{2}$, and the total deflection angle: $\Delta =\sum_{j=1}^{p}\left(i_{1}^{\left(j\right)}+r_{2}^{\left(j\right)}-r_{1}^{\left(j\right)}-i_{2}^{\left(j\right)}\right)$.

The dispersion rate is still defined as $\partial F^{\left(p\right)}/\partial \lambda$. However, there are too many influencing factors to derive a direct expression. From the dispersion rate, the resolution derivation is similar to that in the front. The total transmittance is the product of the transmittance of each optical surface. Thus:

(4) $$T^{\left(p\right)}=\prod_{j=1}^{p}\left(\begin{array}{l} \left(1-\left({\left(\left(\sin(i_{1}^{\left(j\right)}-r_{1}^{\left(j\right)})\right)/\left(\sin(i_{1}^{\left(j\right)}+r_{1}^{\left(j\right)})\right)\right)}^{2}+{\left(\left(\tan(i_{1}^{\left(j\right)}-r_{1}^{\left(j\right)})\right)/\left(\tan(i_{1}^{\left(j\right)}+r_{1}^{\left(j\right)})\right)\right)}^{2}\right)/2\right)\\ {}*\left(1-\left({\left(\left(\sin(i_{2}^{\left(j\right)}-r_{2}^{\left(j\right)})\right)/\left(\sin(i_{2}^{\left(j\right)}+r_{2}^{\left(j\right)})\right)\right)}^{2}+{\left(\left(\tan(i_{2}^{\left(j\right)}-r_{2}^{\left(j\right)})\right)/\left(\tan(i_{2}^{\left(j\right)}+r_{2}^{\left(j\right)})\right)\right)}^{2}\right)/2\right) \end{array}\right)$$

However, although this model can accurately determine the position of the light eventually irradiated on the receiving surface and can further deduce the evaluation parameters, it cannot directly guide the design. The reason is that there are too many parameters involved in this model, and the form of evaluation parameters derived is too complex. When the design parameters are optimized, there may be a complex interaction between different parameters. In this case, simple single-parameter optimization often fails to obtain the optimal design results, and the overall optimization needs to be carried out in the high-dimensional parameter space.

Therefore, we directly constructed a new holistic and generalized model and then optimized the model. Firstly, we analyzed the nature of dispersion of multiple prisms (prisms). Prism dispersion, or the material dispersion method, is essentially achieved using different propagation velocities of light at different wavelengths in matter, or different refractive indices. Light of different wavelengths will propagate in different directions. The prism of matter essentially makes use of the interface of two different mediums, which makes the direction of light propagation of different wavelengths differ from each other due to the differences in the amount of variation. Then, using the distance between the interfaces, the angle difference can be transformed into a space distance to achieve the effect of dispersion.

We attempt to describe this new model in mathematical language. First, we discuss two specific wavelengths of light passing through the entire prism group, which is equivalent to passing through several dielectric interfaces. Suppose that after passing through the interface of the *j*th medium, the angle at which the two wavelengths of light are separated is θjΔ, and the distance between the *j*th interface and the (*j* + 1)th interface is $d_{j}$. After the first interface and in the interval between the first surface and the second surface, the distance between the two wavelengths of light is θ1Δd1. After the second interface and in the interval between the second plane and the third plane, the distance between the two wavelengths of light is θ1Δ(d1+d2)+θ2Δd2. By analogy, if there are *P* interfaces in total, the distance between the two wavelengths of light is θ1Δ(d1+d2+……+dp)+θ2Δ(d2+d3+……+dp)+……+θpΔdp = d1θ1Δ+d2(θ1Δ+θ2Δ)+……+dp(θ1Δ+θ2Δ+……+θpΔ).

Next, we need to introduce a design assumption (design constraints), i.e., in the design of dispersion schemes, the total path length that light can pass through $d_{1}+d_{2}+\ldots \ldots +d_{p}=D$ is certain. The miniaturization of dispersion structures is necessary for a compact flow cytometer system. Under this design constraint, in order to maximize the “distance from which light is separated”, each *d* (which represents any $d_{j}$) is essentially allocated. According to the above deduction, we can see that for different values of *d*, the coefficients multiplied ahead are different. Each θΔ (which represents any θjΔ) should be non-negative. Therefore, the coefficient multiplied ahead, $d_{p}$, is the largest. To maximize the linear dispersion rate, $d_{p}$ should be maximized. In addition, a generalized inference can be drawn; it is better to set $d_{p}$ larger, rather than set $d_{p-1}$ or another *d* value. Thus, the greatest result of d1θ1Δ+d2(θ1Δ+θ2Δ)+……+dp(θ1Δ+θ2Δ+……+θpΔ) is D(θ1Δ+θ2Δ+……+θpΔ). However, in practice, we cannot let other *d* = 0, so there will be some deviations in the results.

This part of the analysis follows the following ideas. First, the direction of the optical path should be determined, and then the prism is matched according to the direction of the optical path so that the optical path can be formed eventually. The angle arrangement and distance arrangement of the prism are determined independently. According to the analysis in Section 2.1, the optimal single prism design is unique; thus, all prisms are the same. On the premise that only the general plane beam is considered, the total deflection angle, Δ, of the light would not exceed 180 degrees. Therefore, the number of prisms $N=\frac{\Delta }{\delta }$ (round down) can be determined according to the deflection angle, *δ*, of a single prism, which is decided by *α* and $i_{1}$.

Thus, we can directly analyze the parameters $T^{N}$, N(∂k/∂n), and $N(\partial r_{2}/\partial n)$ under the superposition of multiple prisms in a similar way to that in Section 2.1. For example, we calculated the optimum value $N(\partial r_{2}/\partial n)$ under different lower transmittance limits and the values of the top and incidence angles when using the results from Figure 2d and the data of all $N(\partial r_{2}/\partial n)$. After determining the number of prisms according to the result of *α* and $i_{1}$ chosen in Figure 2d, the shape of the optical path is essentially determined. For example, for four prisms, this means that the light path turns four times within 180 degrees of total rotation. This is the shape of half an octagon.

Therefore, the prism design process can be summarized as follows:

1. Identify at least what transmittance is required;
2. According to the refractive index and the process above, obtain the optimum apex angle, incidence angle, and the number of prisms. The number of prisms determines the shape of the regular polygon along which the light path moves;
3. Determine the size (radius) of the optical path polygon according to the size limitation of the system (or the desired size);
4. According to the results of apex angle and incidence angle, prisms are arranged at each vertex of the polygon. The size of the prism is only larger than the width of the input light (avoid light loss), and the apex angle of the prism is as close as possible to the light path (leaving a certain margin).

## 3. Results

### 3.1. Design and Spectral Calibration

**Figure 3:**
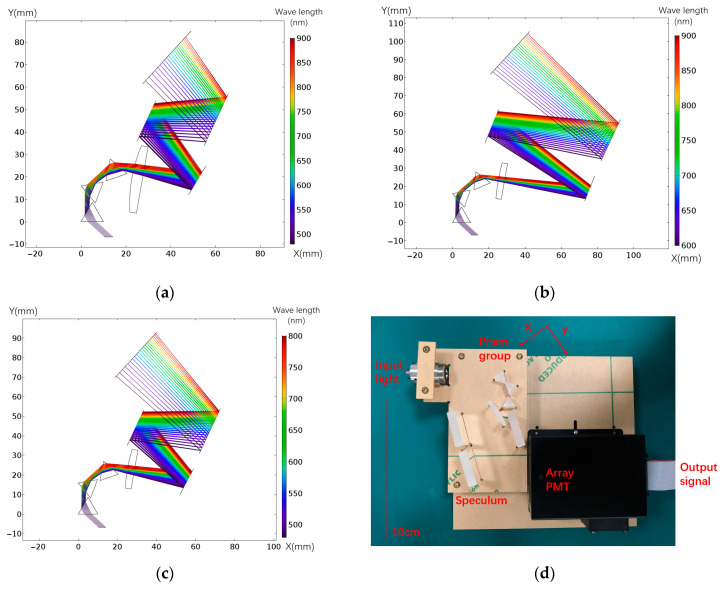

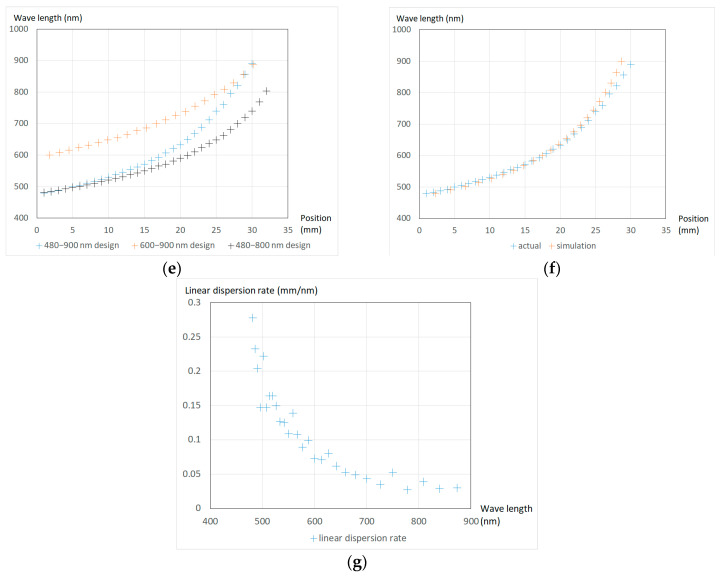
Prism design examples and spectral calibration (simulation performed with COMSOL Multiphysics): (**a**) the design of 480−900 nm; (**b**) the design of 600−900 nm; (**c**) the design of 480−800 nm; (**d**) hardware system of design in (**a**); (**e**) all of the spectral calibration results of the designs; (**f**) experimental spectral calibration results of design in (**a**), and the comparison with the simulations; (**g**) experimental linear dispersion rate results of design in (**a**).

According to the above design process, we designed the prism structure for fluorescence spectrum detection. In Figure 3, three design examples are shown when the top angles are 60 degrees, including the spectral calibration results: the general 480−800 nm design, the long wave enhanced 480−900 nm design, and the 600−900 nm design. These designs are simulated in COMSOL Multiphysics, which is the software from COMSOL (Stockholm, Sweden). In these three designs, the incidence angles of each prism are 47.19, 47.46, and 47.46 degrees, respectively. The side length of the prisms is 10 mm, which is used as the standard to adjust the size of the light path polygon so that the prisms do not interfere. For different dispersive bands, it is necessary to adjust the prism position so that light in a certain wavelength range falls in the detector’s location and then completes the follow-up function. Therefore, this design theory has the advantage of flexibility for different spectral bands. Figure 3e shows the spectral calibration results of different designs. These results are measured with a monochromator. Figure 3f compares the result with the simulation in Figure 3a. The simulation results show that 480–900 nm is distributed at the position of 2.2–28.7 mm, and the actual distribution is at the position of 1.0–30.3 mm. The dispersion length of the actual result is 111% of the simulation result. In these results, Figure 3g shows that the line dispersion rate of the short wavelength part is higher. This point is more consistent with the fluorescence spectral band distribution of fluorescent markers commonly used in flow cytometry experiments, which can achieve better detection results.

**Figure 4:**
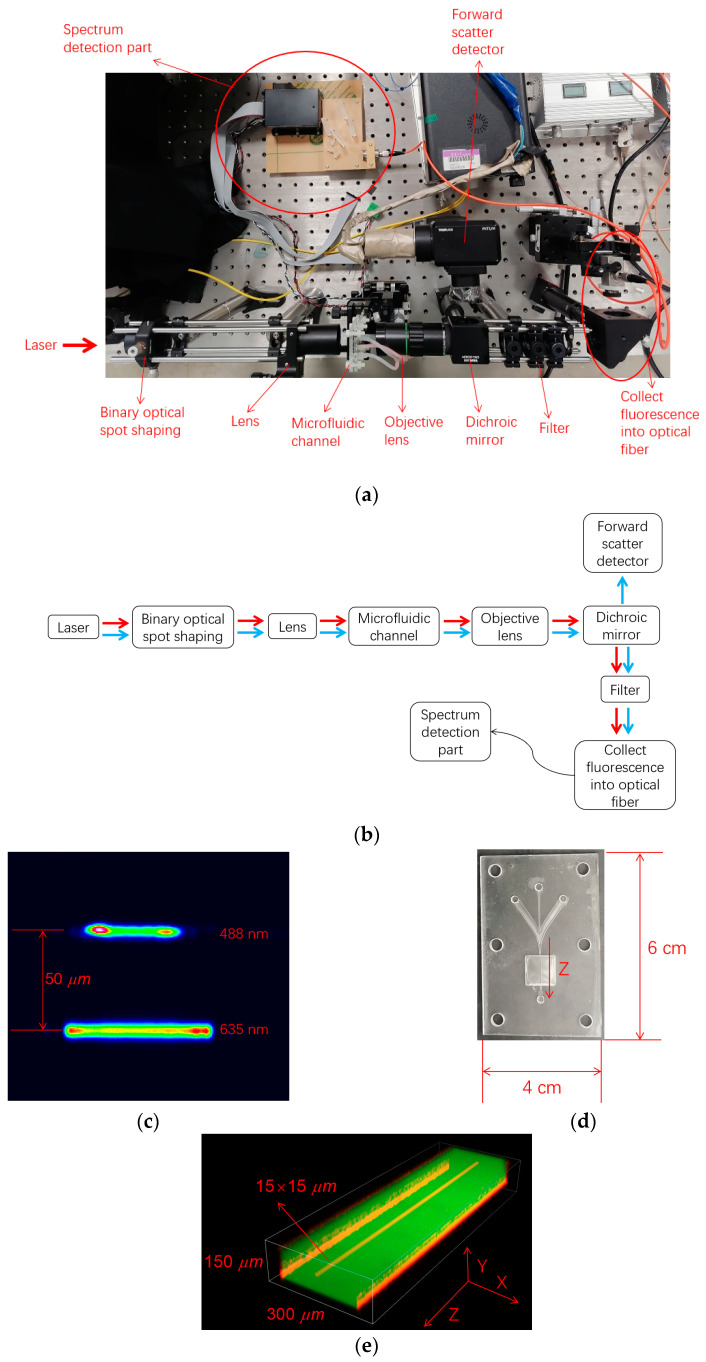
Experimental platform: (**a**) the overall structure (material object); (**b**) the overall structure; (**c**) the shaped laser spots; (**d**) microfluidic chip for hydrodynamic focusing; (**e**) in the microfluidic chip, the red sample flow is focused at the center and the green is sheath; flow velocity is 5 m/s.

According to Figure 4a, the dispersion part is connected to the micro-flow cytometer experimental platform for testing. The hardware used in the system included the array PMT H11460-01 from Hamamatsu Photonics (China) Co. Ltd. (Beijing, China), and the THP1830_SCH multi-channel data acquisition system customized by Beijing Huaqing Ruida Technology Co. Ltd (Beijing, China). The optical elements used in the optical path system of the prism part are all from Thorlabs Inc (Shanghai, China). These include the prism PS850, the cylindrical lens LJ1934L1-A, and the reflection mirrors BBSQ1-E02. The flow cell experiment platform and the prism dispersion part conduct light through optical fibers. As shown in Figure 4a, the two-color laser spot is shaped by binary optical elements, and the microchannel on the chip, which is shown in Figure 4d, is used as the liquid path. The shaped light spot is depicted in Figure 4c. Confocal microscopy was used to observe the converging effect of flow passage on liquid flow, as shown in Figure 4e [20,21,22,23,24].

### 3.2. Microsphere Experiment

**Figure 5:**
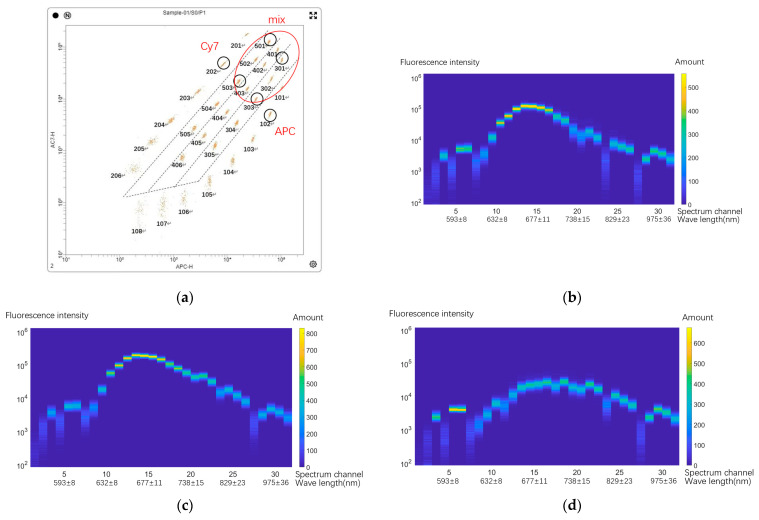

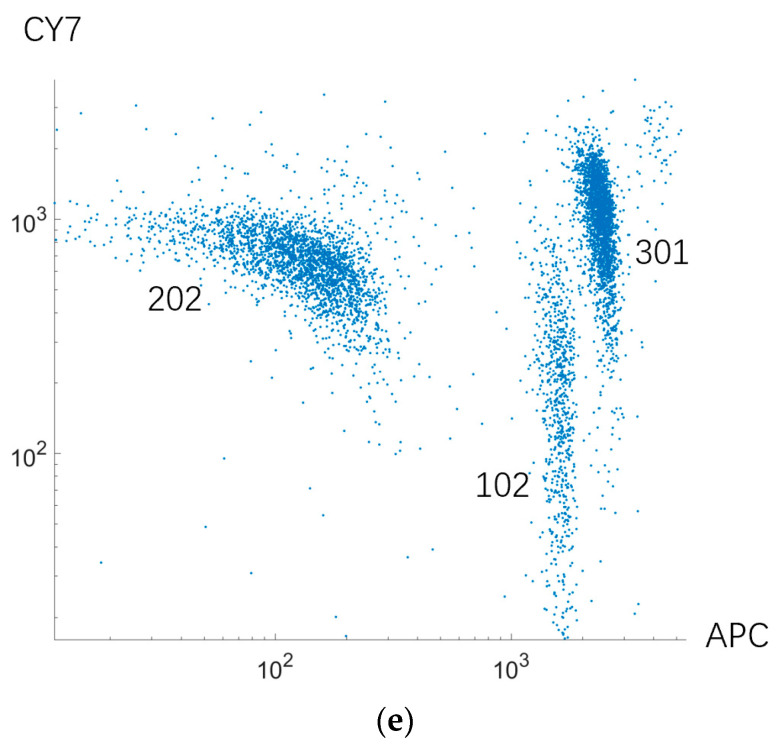
Microsphere experiment: (**a**) fluorescence information of experimental samples used (this figure was provided by Shenzhen Wellgrow Technology Co. Ltd.); (**b**) the raw spectral data of ‘102’ tested by our system; (**c**) the raw spectral data of ‘301’ tested by our system; (**d**) the raw spectral data of ‘202’ tested by our system; (**e**) microsphere experiment result after comprehensive spectral calculation.

We used microspheres containing different kinds of fluorescent substance to verify the overall detection and spectral solution capabilities of the system. The fluorescence wavelength of the fluorescent substance used here is different from that of the fluorescent marker used in the subsequent cell experiment, which is only used as an analog verification of the principle and method. In this experiment, the microspheres used were fluorescent coded microspheres produced by Shenzhen Wellgrow Technology Co. Ltd. (Shenzhen, China). Fluorescent coded microspheres contain two fluorescence dyes. Primarily, they exhibit differences between series, and different series of fluorescent coded microspheres contain different kinds of fluorescent substances. In the same series, the fluorescent substances of different types of fluorescent coded microspheres have the same composition but different contents. Secondly, in addition to the single type of coded microspheres, there are coded microspheres containing two different fluorescent substances mixed in specific proportions. The functional verification of the system can be achieved by combining these different microsphere models. Figure 5 depicts these details.

In Figure 5a, the series of microspheres starting with ‘10’ are pure APC (Allophycocyanin) fluorescent microspheres. The microsphere series starting with ‘20’ is pure Cy7 (Sulfo Cyanine 7, an anthocyanin, coupled by APC, shown as ‘AC7’ in the figure, so ‘Cy7’, ‘APC-Cy7’ and ‘AC7’ are the same things) fluorescent dye. Figure 5a is the original channel intensity map without compensation adjustment, which was provided by Shenzhen Wellgrow Technology Co. Ltd. The reason these two-dye series are not arranged along the horizontal or vertical coordinates in the above figure is a problem of the spectral leakage of fluorescence. APC has a signal in the wavelength channel of Cy7, and Cy7 also has a signal in the spectral channel of the wavelength of APC. Therefore, the two series are arranged diagonally at different positions in the figure. The various series in the middle of them are microspheres formed by mixing the two dyes in different proportions on the same microspheres. Each microsphere contains different proportions of these two dyes. For example, 301 and 303 in the above figure have the same mixing ratio of APC and Cy7; only the total intensity of fluorescent dye on the two types of microspheres is different, as shown in Table 1. The contents of APC and Cy7 contained in each kind of microsphere can be measured through spectral detection, and the influence of spectral leakage can be overcome. It is important to test the function of the system.

In the actual experiment, as described above, the single fluorescent material microspheres of 102 and 202 were first measured as the base of the fluorescence spectrum, and the models of these two microspheres were directly used to represent fluorescein in the subsequent analysis; then, the mixed fluorescent microspheres 301 and 501 with different mixing ratios were measured to verify the ability of spectral measurements and calculations. Some of the raw spectral data are shown in Figure 5b–d, corresponding to microspheres 102, 301, and 202. Each experiment ensured that the effective data volume of each kind of microsphere was not less than 5000; the average value of the population was taken for calculation. The non-negative least-squares method based on principal component analysis was used to solve the specific fluorescence content data. The results are presented in Table 2.

After repeated experiments, it can be seen that the random error of the resolution result of fluorescein content is not more than 5%, which has good uniformity. The experimental microsphere results after comprehensive spectral calculations are shown in Figure 5e. The actual performance of the system is also limited by the flow channel, optical path, and other parts not studied in this article. Therefore, this system has a detection capability similar to that of ordinary flow cytometer instruments.

### 3.3. Cell Experiment

**Figure 6:**
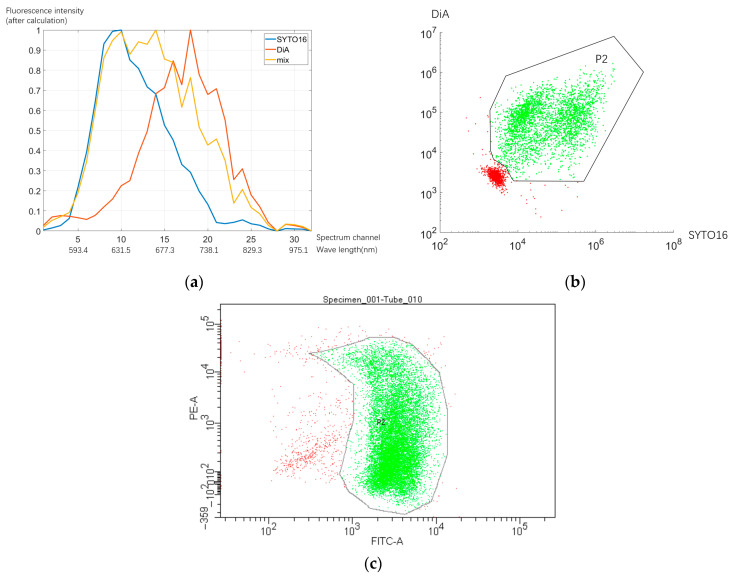
Cell experiment: (**a**) spectral results of different samples (our system); (**b**) flow cytometry results for double-staining samples in our system; (**c**) flow cytometry results for double-staining samples in BD LSRFortessa.

In flow cytometry, the detection of cells is indirectly realized by the detection of different types of fluorescent markers on cells. In order to verify the spectrum measurement of the system for fluorescent markers with different wavelengths, HeLa cells were used as cell samples for experiments, and two fluorescent dyes with different wavelengths—SYTO16 nucleic acid dye obtained from Thermo Fisher, and DiA (Pyridinium,4-[2-[4-(dihexadecylamino)phenyl]ethenyl]-1-methyl-, iodide (1:1)) cell membrane dye from Shanghai Biyuntian Biotechnology Co. Ltd. (Shanghai, China)—were used to label cells. The cells obtained from the same batch of passages were processed into three batches of samples: SYTO16 single staining, DiA single staining, and two simultaneous double staining. Then, our system and BD LSRFortessa flow cytometer were used to analyze these three batches of samples; the results are depicted in Figure 6.

Figure 6a indicates that the system can obtain different spectral data measurement results for three batches of different cell samples. The average mixing ratio of the two dyes in the mixed staining cells is 0.8824:0.5154 (1:0.5841). The scatter diagram of the two fluorescein components of each particle for double-staining samples after comprehensive spectral calculation is shown in Figure 6b. These results are compared with those of BD LSRFortessa in Figure 6c.

In our spectral flow system, the coefficient variation of SYTO16 was 132.2%, and DiA was 75.0%. For BD LSRFortessa, because it belonged to traditional flow cytometry, it could only be analyzed using fixed spectral channels. According to the spectral distribution, syto16 was analyzed using the fluorescein isothiocyanate (FITC) channel, and DiA was analyzed using the p-phycoerythrin (PE) channel. The coefficient variation of the FITC channel was 46.3%, and that of the PE channel was 238.6%. The spectral flow cytometer could produce more concentrated results, i.e., closer to a circular shape. 

## 4. Discussion and Conclusions

This paper introduced the basic optical theory, based on the case of single prism, and considered the case of multi-prism. The sizes required for miniaturization, linear dispersion rate, and light energy utilization rate were the design objectives of this study. Finally, a set of prisms was provided to complete the fluorescence detection function of the spectral flow cytometer, which was applicable to the design theory of the miniaturization system.

In existing spectral flow cytometers or spectral instruments, the grating scheme is widely used. In order to compare with our prism study, considering that other studies rarely disclose specific indicators in this area, this section presents commercial grating devices and measures of their indicator data.

**Figure 7:**
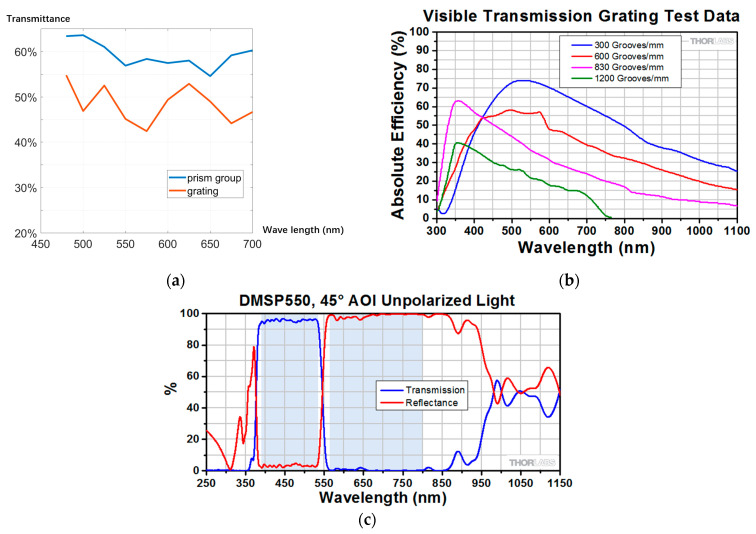
Comparison of light energy utilization of different schemes: (**a**) comparison of experimental results of light energy utilization between prism group (our research) and grating; (**b**) theoretical problems of light energy utilization of grating (this figure was provided by Thorlabs); (**c**) light energy utilization of dichroic mirror (this figure was provided by Thorlabs).

This section details the grating of general commercial products that may be used in existing spectral instruments for comparison (Thorlabs GT13-06V). The specific test method is as follows: use an optical power meter to measure the single wavelength light output by the monochromator and the optical power of the single wavelength light after passing through the prism or grating. The light energy utilization ratio is equal to the ratio of the output light power to the input single wavelength light power. The specific test results are shown in Figure 7.

The test results presented in Figure 7a are consistent with the previous theoretical analysis results, and the prism dispersion scheme had the advantage of higher and more uniform light energy utilization than the grating scheme. The reason for this phenomenon lies in the grating dispersion method itself. According to the general design of blazed gratings, the blaze angle of a grating is bound to a specific angle or wavelength. If the wavelength deviates from the flare angle, the light energy will be greatly lost [25]. Although reducing the reticle density (increase the grating constant) can alleviate this phenomenon, a spectral flow cytometer needs wide band applications. The problem is less serious when the grating constant is large, but the dispersion rate is insufficient, leading to large system size. Therefore, the grating dispersion method is commonly used in general spectral instruments but is not suitable for spectral flow instruments. In comparison, the light energy utilization of our study was more than 50% and had good uniformity.

As for the comparison with other dispersion schemes, the theoretical analysis of the multi-prism scheme is detailed in Section 2.2. The optimal number of prisms is three or four. For a scheme with more than ten prisms, the line dispersion rate cannot be improved theoretically, and the light energy utilization rate is worse (less than 50% theoretically). Using only a single prism can achieve a slightly higher light energy utilization rate, but the role dispersion rate will be significantly reduced. In order to still match the array PMT, it is estimated that the optical path size will be more than 30% larger than the result of this subject (there has been no further research on such schemes in recent years). The actual long side size of other schemes is 15 cm or more, according to the estimated results. The method of this study focused on reducing the volume, which can be reduced by more than 30%.

For the Cytek filter scheme, there is mainly a problem of light energy utilization. According to the average reflectivity of 95% of the common band of the commercial dichroic mirror (taking Thorlabs DMSP550 in Figure 7c as an example), in the case of 32 channels, the light energy utilization rate after 31 reflections was lower than 30%, and the uniformity of each channel was poor. Thus, a stronger photoelectric detection capability is required to bring greater indicator pressure to the subsequent links. All of these are compared in Table 3.

Compared with the existing design of a spectral flow cytometer based on prism dispersion, this design can achieve the smallest size and the highest light energy utilization. Compared with the gratings for dispersion, its advantages are mainly as follows:

- Higher light energy utilization. The light energy utilization rate of this design can exceed 50% under appropriate parameter selection, which is difficult to achieve through grating dispersion, especially with a wavelength deviating from the blaze angle;
- More uniform light energy utilization of each wavelength. Prism dispersion does not have the blaze angle problem, and more comprehensive spectral information can be obtained while improving light energy utilization;
- Higher dispersion rate at short wavelengths. In flow cytometry, 480−600 nm is the most frequently used wavelength with the largest amount of spectral information. The property of prism dispersion makes the linear dispersion rate of this part of wavelength larger than that of the whole, which improves the measurement accuracy of this part of information.

At the same time, there are still some shortcomings in this design. The design and optimization of grating dispersion are not discussed in this paper. However, according to the discussion in this paper and some other articles, the light energy utilization ratio of grating is low, and the prism dispersion scheme has the advantage of a high light energy utilization ratio compared with the grating scheme [14,26]. However, the grating scheme has other advantages, such as fewer optical elements. Considering the need for miniaturization, it is also a desirable solution.

In addition, this research only focused on the design of hardware structures. In fact, the data solution algorithm is also the key to distinguish a spectral flow cytometer from a traditional flow cytometer [27,28]. In recent years, the optimization of this aspect has gradually become a research hotspot in the field of spectral flow cytometry [29,30]. This paper simply presents a general hardware model design method. On the basis of this study, better results can be achieved by combining the optimization of data solution, which is also the potential application of this study.

In conclusion, the specific design theory proposed in this paper focused on the application of prism dispersion in flow cytometry. Quantitative design theory and design criteria are presented. Follow-up studies could be carried out more efficiently on this basis.

## 5. Patents

Zheng You, Zixi Chao, and Jingjing Zhao. Optical path design method and device for fluorescence dispersion of flow cytometer, CN114136867A[P]. 2021.

Zheng You, Zixi Chao, and Jingjing Zhao. Waveguide design for on-chip fluorescence dispersive optical path for flow cytometer, CN114112873A[P]. 2021.

## Figures and Tables

**Table 1:** Composition of fluorescent substances in different types of microspheres.

Type	APC	Cy7
102	Contain	Not contain
202	Not contain	Contain
301, 501	Contain	Contain

**Table 2:** Spectral experimental results of microspheres.

Experiment	Sample	102 Content	202 Content	Relative Mixing Ratio
Mix ratio test for 301	102	1	0	
	202	0	1	
	301 (first experiment)	1.7247	1.1902	1.449
	301 (second experiment)	1.6992	1.2255	1.387
	301 (third experiment)	1.7405	1.1993	1.451
Standard deviation/mean		0.0099	0.0124	0.0208
Mix ratio test for 501	102	1	0	
	202	0	1	
	501 (first experiment)	0.2936	2.7813	0.106
	501 (second experiment)	0.2979	2.5614	0.116
	501 (third experiment)	0.2915	2.5869	0.113
Standard deviation/mean		0.0091	0.0372	0.0375

**Table 3:** Comparison of indicators between our design and other designs.

Institution and Year	Dispersion Method	Detector	Size of Dispersion Part (Long Edge) *	Spectral Range	Average Linear Dispersion Rate and Distribution *	Detection Flux
Tsinghua University, 2022 (our study)	Prism group	Array PMT	Less than 10 cm	480–900 nm	0.10 mm/nm Higher in short wavelength	More than 5000 cells/s
Cytek Biosciences, 2017	Light filter	Coarse wavelength division multiplexing array	Approximately 10 cm	365–829 nm	Higher in short wavelength (specific data not disclosed)	20,000 cells/s
Kyoto University, 2015	Prism group	Array PMT	Approximately 33 cm	500–800 nm	0.10 mm/nm Higher in short wavelength	20,000 cells/s
Purdue University, 2012	Grating	Array PMT	Approximately 15 cm	525–767 nm	0.13 mm/nm Basically uniform	3000 cells/s
Los Alamos National Laboratory, 2006	Grating	Array CCD	Approximately 15 cm	400–800 nm	0.010 mm/nm Basically uniform	42 cells/s
Macquarie University, 1996	Single prism	Array photodiode	Approximately 19 cm	488–800 nm	0.020 mm/nm Higher in short wavelength	62.5 cells/s

* These data in this field are not directly disclosed in such studies; therefore, we present results of our estimation or theoretical derivation with reference to existing materials.

## Data Availability

Not applicable.
